# Hypertension and Cognitive Impairment: A Review of Mechanisms and Key Concepts

**DOI:** 10.3389/fneur.2022.821135

**Published:** 2022-02-04

**Authors:** Michelle Canavan, Martin J. O'Donnell

**Affiliations:** ^1^Health Research Board (HRB), Clinical Research Facility, National University of Ireland, Galway, Ireland; ^2^Galway University Hospital, Galway, Ireland

**Keywords:** hypertension, cognitive impairment, dementia, neurocognitive syndrome, vascular cognitive impairment and dementia (VCID), blood pressure lowering

## Abstract

Cognitive impairment, and dementia, are major contributors to global burden of death and disability, with projected increases in prevalence in all regions of the world, but most marked increases in low and middle-income countries. Hypertension is a risk factor for both Vascular Cognitive Impairment and Alzheimer's disease, the two most common causes of dementia, collectively accounting for 85% of cases. Key end-organ pathological mechanisms, for which hypertension is proposed to be causative, include acute and covert cerebral ischemia and hemorrhage, accelerated brain atrophy, cerebral microvascular rarefaction and endothelial dysfunction, disruption of blood-brain barrier and neuroinflammation that affects amyloid pathologies. In addition to the direct-effect of hypertension on brain structure and microvasculature, hypertension is a risk factor for other diseases associated with an increased risk of dementia, most notably chronic kidney disease and heart failure. Population-level targets to reduce the incidence of dementia are a public health priority. Meta-analyses of blood pressure lowering trials report a significant reduction in the risk of dementia, but the relative (7–11%) and absolute risk reductions (0.4% over 4 years) are modest. However, given the high lifetime prevalence of both conditions, such relative risk reduction would translate into important population-level reductions in dementia globally with effective screening and control of hypertension. Optimal blood pressure target, especially in older adults with orthostatic hypotension, and antihypertensive agent(s) are uncertain. In this review article, we will detail the observational and interventional evidence linking hypertension with cognitive impairment, summarizing the mechanisms through which hypertension causes cognitive decline.

## Introduction

**Figure 1:**
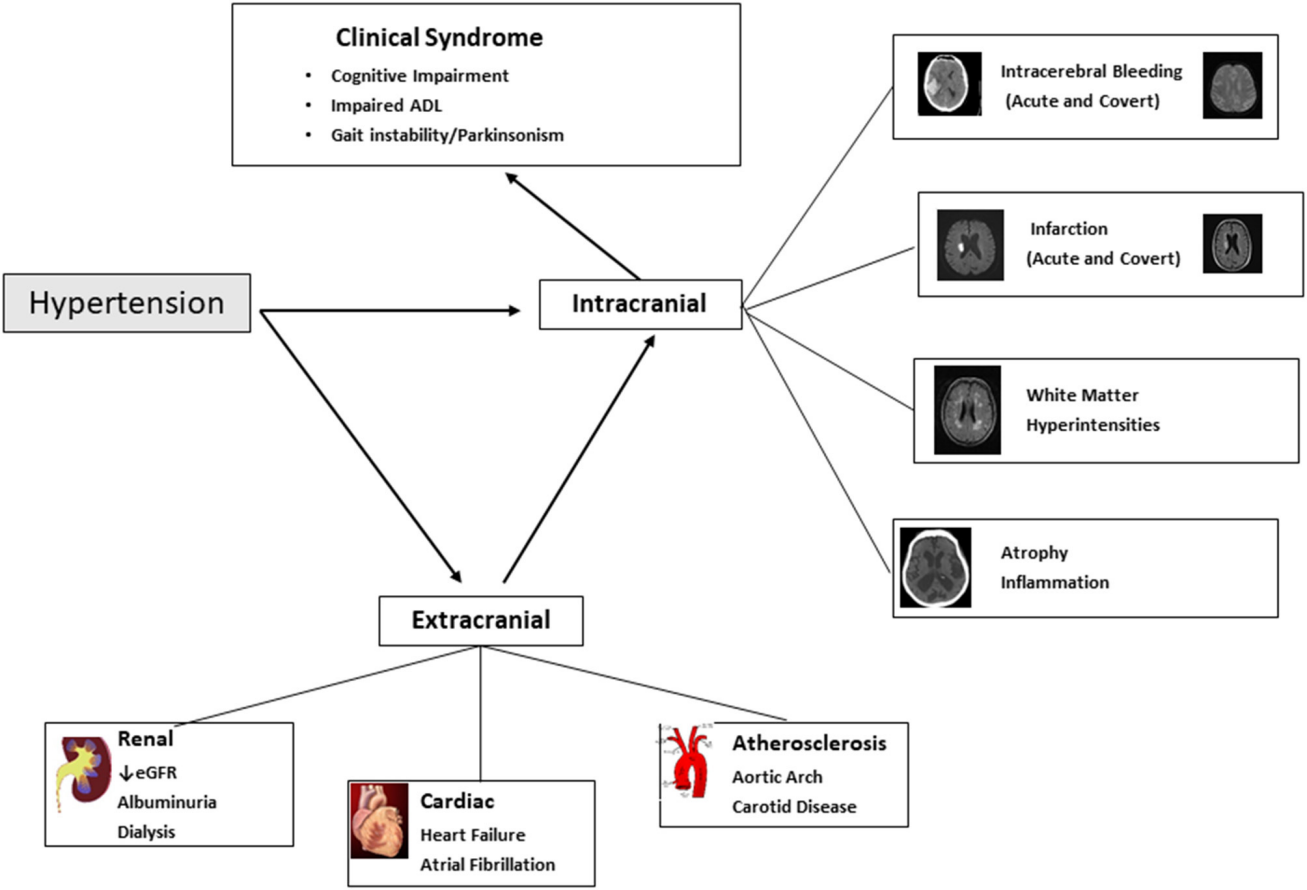
Hypertension and cognitive impairment and dementia. Figure illustrates the intracranial and extracranial mechanisms through with hypertension results in cognitive impairment and dementia (and illustrates other non-cognitive clinical consequences that are part of the dementia syndrome). ADL, Activities of Daily Living; eGFR, Estimated Glomerular Filtration Rate.

Hypertension causes acute and chronic injury to the brain, accelerates brain atrophy and engages neuroinflammatory processes, each of which contribute to cognitive impairment and major neurocognitive syndromes (dementia) (1). In addition to a “direct-effect” of hypertension on brain structure and microvasculature, hypertension is a risk factor for other syndromes related to end-organ damage, which are also associated with an increased risk of dementia, most notably chronic kidney disease and heart failure (Figure 1).

Hypertension is a risk factor for both Vascular cognitive impairment and Alzheimer's disease, the two most common etiologies of dementia which commonly co-exist, and collectively account for 85% of cases of dementia (2). At present, there are no widely available effective treatments that favorably alter the natural history of cognitive decline and dementia, placing enhanced emphasis on the importance of primary prevention (3).

Identification and treatment of hypertension is considered an important target for population-level reduction in global burden of dementia (4). Although there have been improvements globally in detection of hypertension, levels of treatment and control are variable with one study quoting control rates of 23% for women and 18% for men in 2019 with worse rates in low to middle income countries where there is increasing prevalence of hypertension. Unequal access to medications, universal health- care and low levels of implementation of targeted public health measures may account for the low rates of control which will ultimately increase the burden of hypertension related conditions including ischemic heart disease and heart failure, chronic kidney disease and dementia (5).

**Figure 2:**
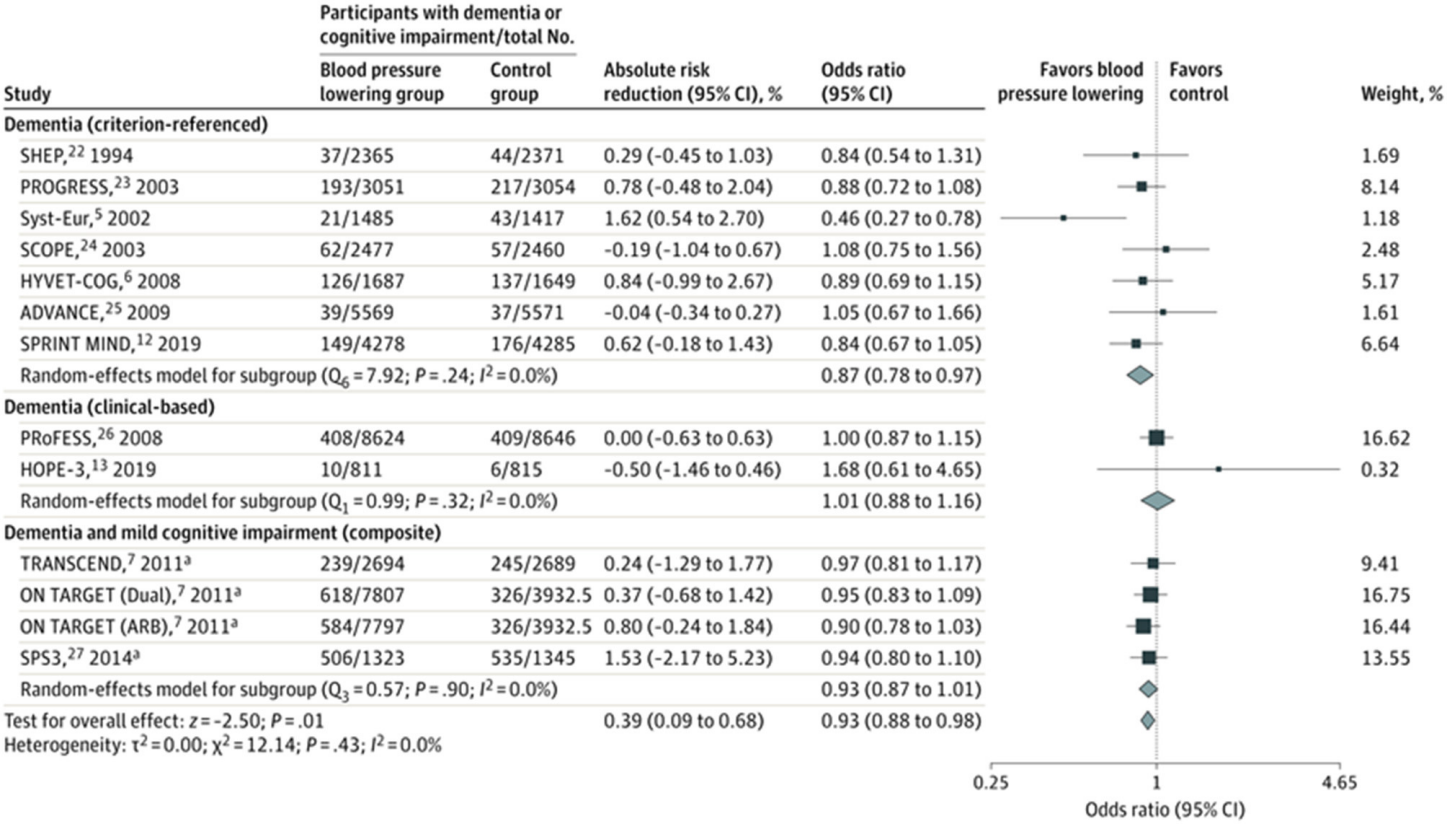
Meta-analysis of blood pressure lowering randomized controlled trials. The squares and bars represent the mean values and 95% CIs of the effect sizes and the area of the squares reflects the weight of the studies. Diamonds represent the combined effects and the vertical dotted line represents the line of no association. ^a^Composite of dementia and cognitive impairment [Hughes et al. (6), reproduced with permission].

However, while mid-life hypertension increases the relative risk of life-time dementia by 20–54%, use of antihypertensive therapy is associated with a more modest reduction in risk of dementia, with recent meta-analysis of trials reporting 7–11% relative risk reduction (6) (Figure 2). Nonetheless, such a relative risk reduction in prevalence of dementia among individuals with hypertension constitutes a considerable reduction in absolute frequency of dementia globally, given the high lifetime prevalence of both conditions (7).

In the following review article, we will detail the observational and interventional evidence linking hypertension with cognitive impairment, summarize the mechanisms through which hypertension causes cognitive decline, and explore some of the key unanswered questions in the field.

## Epidemiology

### Hypertension Is a Risk Factor for Cognitive Impairment and Dementia

Prospective cohort studies mostly report a positive association of hypertension and risk of cognitive impairment and dementia (8–12), with the strongest association between mid-life hypertension and risk of future cognitive decline and incident dementia. A recent meta-analysis of observational studies, including data from 135 prospective cohort studies (three of which employed nested designs) with over 2 million individuals, reported a significant association of mid-life history of hypertension (RR 1.20; 1.06–1.35), elevated systolic blood pressure (RR 1.54; 1.25–1.89) and diastolic blood pressure (RR 1.50; 1.04–2.16) with risk of dementia. In that analysis, an increased risk emerged with systolic blood pressure over 130 mmHg (1). Among participants in later life, they did not report an overall association of hypertension with dementia risk, but did find a significant association for progression from mild cognitive impairment to dementia (RR 1.41; 1.00–1.99). In contrast to mid-life blood pressure, the risk of dementia emerged with systolic blood pressure over 180 mmHg (RR 1.45; 1.03–2.06). In older age groups, there was an apparent protective effect of diastolic blood pressure with dementia (RR 0.77; 0.59–1.00 for diastolic blood pressure of 90 mmHg or greater), likely reflecting the emergence of competing blood pressure mechanisms (e.g., orthostatic hypotension) (see below). A feature of these, and other analyses was apparent heterogeneity by ethnicity, with higher risks in older adults reported among black populations, compared to other ethnicities (13).

### Population Attributable Fraction (Hypertension and Dementia)

Livingston et al. reported the PAF associated with common risk factors, based on meta-analytic estimates from observational studies, and reported a PAF of 5.1% (2.9–3.6%) and weighted PAF of 2.0% (0.6–0.9%) (14). In contrast, the reported PAF for acute stroke is estimated to be 49–64% (15, 16). However, PAF estimates for dementia were based on a prevalence of hypertension of 8.9%, which is expected to be an underestimate. The 10/66 Dementia Research Group (*n* = 12,865) (14) reported the cross-sectional association of mid-life hypertension, reporting a PAF of 18.6% for China, 25% for South America and 10.4% for India. These estimates are based on self-reported hypertension, and PAF would be larger when accounting for undiagnosed hypertension.

Given these considerations, it is anticipated that the PAF related to hypertension, especially in regions where rates of identification and control are low, particularly LMICs, will be where the largest burden of dementia will be borne. Moreover, the PAF does not account for the intermediary risk factors (e.g., atrial fibrillation) and chronic diseases (e.g., chronic kidney disease and heart failure), which also contribute to the global burden of cognitive impairment and dementia (17). Determining the PAF related to hypertension for dementia is also affected by outcome ascertainment of dementia which varies hugely between studies and sometimes focuses only on cognitive test scores which may not reflect the level of functional decline which is the core part of diagnosis in real world clinical practice.

## Pathophysiology (Mechanisms and Mediators of Risk)

As detailed in Figure 1, there are a myriad of causal pathways through which hypertension can contribute to adverse structural and functional consequences on the brain, leading to development and progression of cognitive decline.

### Structural Changes in the Brain

Key end-organ pathological mechanisms, for which hypertension is proposed to be causative, include acute and covert cerebral ischemia and hemorrhage, accelerated brain atrophy, cerebral microvascular rarefaction and endothelial dysfunction, disruption of blood-brain barrier and neuroinflammation that affects amyloid pathologies (18) (Figure 1).

#### Cerebral Ischemia

Acute ischemic stroke and transient ischemic attack (TIA) are associated with an increased risk of cognitive impairment and dementia. A meta-analysis of population-based studies reported a rate of 7.4% (within 1 year) in population-based studies of first stroke without a history of dementia, and prevalence of 41.3% (95%CI 29.6–53.1%) in hospital-based studies of recurrent stroke (19). Hypertension is also associated with covert brain infarction, i.e., present on neuroimaging but without an acute clinical presentation of stroke, which are most often (90%) discrete small infarcts located in white matter or subcortical structures in the brain (Figure 1).

Beyond infarction, hypertension may be considered as an accelerator of aging cerebral vasculature, especially for small vessel disease. Small vessel disease is also a risk factor for post-stroke cognitive impairment (20). The effect of hypertension on small vessels within the brain can related to endothelial damage, lipohyalinosis, fibrinoid necrosis, microaneurysms, and pericyte injury. In addition, hypertension can result in reduced blood flow through a process of rarefaction, which has been demonstrated in animal models in renal and cerebral vascular beds. One common manifestation is white matter hyperintensities (Figure 1), which involve the coalescence of hyperintense signals in the periventricular structures of the brain, and their presence is associated with an overall 2-fold increase in dementia (21), but risk is related to burden of hyperintensities (22). Moreover, severity of white matter hyperintensity is associated with loss of instrumental activities of daily living (e.g., looking after finances), meaning that individuals are more likely to be diagnosed with dementia, which requires the combination of cognitive deficits with attributable impairment in activities of daily living (23).

#### Cerebral Hemorrhage

Cognitive impairment is common after acute intracerebral hemorrhage, for which hypertension is the dominant risk factor, with prevalence ranging from 19 to 63% at 6 months after intracerebral hemorrhage (24). Similar to ischemic stroke, an important determinant of whether patients develop cognitive impairment is location and size of stroke. In addition to acute hemorrhage, covert cerebral microbleeds, small discrete areas of bleeding (<5 mm diameter) (Figure 1), are also a manifestation of small blood vessel disease, are associated with cognitive decline (25).

#### Brain Atrophy/Inflammation

Hypertension is a risk factor for presence and severity of brain atrophy, a key feature of neurodegenerative diseases. Elevated blood pressure is associated with brain atrophy, and increased number of neuritic plaques in neocortex and hippocampus and neurofibrillary tangles in autopsy studies (26, 27). Other mechanisms include oxidative stress with microvascular damage and inflammation. A proposed mechanism, which promotes inflammation is disruption of the blood brain barrier with microglia activation, and impaired glymphatic clearance of amyloid (28–30). These latter mechanisms likely account for the contribution of hypertension to accelerating Alzheimer's disease mechanisms.

### Extracranial Mechanisms and Mediators

#### Chronic Kidney Disease

Hypertension is a major risk factor for chronic kidney disease. The prevalence of cognitive impairment in people with CKD ranges from 10 to 40% (31, 32), with the highest in those receiving haemodialysis where approximately half of patients undergoing dialysis have moderate to severe cognitive impairment (33). Both reduced estimated glomerular filtration rate and albuminuria are independent risk factors for development of cognitive impairment and dementia (34). The association of albuminuria and cognitive impairment is largely mediated through a common mechanism of vascular endothelial damage. Chronic uraemia is associated with loss of blood brain barrier integrity contributing to cerebral small vessel ischemia (35). In dialysis populations, additional contributors include fluctuating blood pressure during ultrafiltration, an inadequate autonomic response to this fluctuation, as well as cerebral stunning which can cause cerebral injury and hypo-perfusion (36, 37). Other mechanisms by which chronic kidney disease contributes to cognitive impairment include vascular calcification and arteriosclerosis (38). Chronic kidney disease is also a risk factor for acute and covert stroke, independent of hypertension, and has also been associated with increased beta amyloid production and impaired clearance of beta amyloid (39) (Figure 1).

There is some evidence of a shared natural history of disease (40), with hypertension playing an overlapping role in both chronic kidney disease and cerebrovascular disease. Both kidney (afferent arterioles) and brain (deep perforating) arterioles are exposed to high pressure, requiring them to maintain large pressure gradients which make them particularly prone to hypertensive injury and problems with autoregulation (Strain Vessel Hypothesis) (41). There is a long latent period between the damage to the kidney from hypertension and a decline in kidney function similar to the effect of prolonged hypertension on cognition and it may be accelerated by other cardiovascular events (42). In randomized controlled trials of blood pressure lowering, the relative risk reduction in renal outcomes is consistent with estimates for cognitive outcomes, based on indirect comparisons of meta-analyses, and findings from the SPRINT trial (43, 44).

#### Extracranial Large Vessel

Large vessel atherosclerosis is associated with an increased risk of ischemic stroke and increased risk of Alzheimer's disease (45). In addition, hypertension also results in age-related stiffening of the elastic arteries in aortic arch and large vessels, which provide an important buffering role in dampening haemodynamic pulsatility (Windkessel effect), with hypertension resulting in the increased pulsatility pressure in brain. In older adults with hypertension, this increased pulsatile pressure results in greater strain on the cerebral microcirculation (46).

#### Cardiac Disease

Hypertension is an important risk factor for heart failure (RR 1.40; 1.24–1.59), with an estimated PAF of 10.1%, based on analysis of NHANES dataset (47). Heart failure is an independent risk factor for dementia, associated with a 28% relative odds increase in risk. Hypertension is also major risk factor for atrial fibrillation, which, in turn, is associated with an increased risk of cognitive impairment (48), mediated largely though the risk of thromboembolism. In a meta-analysis of 43 cohort studies, atrial fibrillation was associated with a 50% increase in relative odds of cognitive impairment or dementia (OR 1.5; 1.4–1.8).

### Hypertension, Cognitive Domains, and Aetiological Subtypes of Dementia

While hypertension is reported to be a risk factor for Vascular Cognitive Impairment and Alzheimer's disease, there is considerably less convincing evidence of an association with Lewy Body Dementia and Frontotemporal dementia, which occur at lower frequency. In older populations, however, vascular disease commonly co-exists, making it difficult to discern the independence of association between hypertension and neurodegenerative subtypes.

As detailed, the principal mechanisms governing the association of hypertension and cognitive loss are related to vascular disease. Not surprisingly, therefore, hypertension is most strongly correlated with cognitive domains associated with Vascular dementia, but is also a risk factor for global cognition (RR 1.55; 1.19–2.03). For example, one meta-analysis reported a numerically stronger association of hypertension with impairment in executive function (RR 1.22; 1.06–1.41) than memory (RR 1.13; 0.98–1.30), which would be more consistent with Vascular cognitive impairment than Alzheimer's pattern impairment.

Another study reported increased risk in abstract reasoning and executive function loss, which has greater specificity for vascular cognitive impairment (49). People with chronic kidney disease and cognitive impairment also tend to have preferential deficits in executive functioning and processing consistent with the pattern seen in vascular cognitive impairment (50). The pattern of cognitive impairment observed with hypertension or vascular cognitive impairment is often differentiated from the pattern seen in clinical Alzheimer's disease based on preservation of memory function. However, evidence now emerging that hypertension is a risk factor for Alzheimer's Disease by exacerbating accumulation of Aβ in the brain makes it difficult to make a clean distinction and the reality in clinical practice particularly in older people is a mixed picture of both types.

## Does Treating Hypertension Reduce the Risk of Cognitive Impairment?

Recent metanalyses of cohort studies and meta-analyses of randomized controlled trials (13, 51, 52) show a modest benefit of lowering blood pressure on the development of dementia or cognitive impairment. The magnitude of the relative reduction in risk of dementia from blood pressure lowering in clinical trials ranges from 7 to 10% with similar risk reductions noted from observational studies (53). One meta-analysis, that included 96,158 participants from 14 trials, reported an absolute risk reduction of 0.4% (95%CI 0.1–0.7%) in incidence of dementia over a mean follow up of 4.1 years (OR 0.93; 0.88–0.98) (6) (Figure 2). Therefore, the effect size is modest for dementia at an individual-level, but expected to translate into an important population-level impact, with effective identification and treatment of hypertension. In meta-regression, mean age of population (trial-level) was not associated with different treatment effect size, which does not provide support for a differential effect by age.

The relative risk reduction in dementia (7%) associated with antihypertensive therapy is lower than reported for reduction of major cardiovascular events (20%), but similar to the effect reported for renal outcomes (5%) (54). These differential effects of blood pressure lowering on acute cardiovascular events, compared to chronic cognitive and renal outcomes, emphasize that the causative role of hypertension appears to differ by mechanism of disease. In general, large randomized controlled trials of blood pressure lowering are designed to detect treatment effects on incidence of acute events rather than clinical manifestations of vascular disease, which are not event-based. Another distinction is validity and reliability of the outcome measure. Cognitive outcome measures, included in blood pressure lowering trials, range from scores on cognitive testing to centrally adjudicated, criterion-based definitions of dementia. In the SPRINT-Mind trial, they employed a rigorous centrally adjudicated definition of dementia, and reported a 17% relative risk reduction in probable dementia. In one meta-analysis, antihypertensive therapy was also associated with a 13% risk reduction in dementia in clinical trials that employed a criterion-referenced definition of dementia, rather than a clinically defined definition (6). In each of the randomized controlled trials, the cognitive outcomes were secondary or tertiary, and sample size was not based on ability to detect difference in dementia outcomes. Larger trials to determine the effectiveness of multi-domain interventions, targeting a number of cardiovascular risk factors including hypertension, have been completed (55, 56). The largest trial to evaluate the clinical outcome of dementia was the Pre-DIVA trial (56), a cluster randomized trial of older adults in General Practice. A nurse-led cardiovascular risk factor intervention resulted in greater uptake of antihypertensive therapy among those with untreated hypertension at baseline, compared to control (67 vs. 56%). The incidence of dementia after median follow-up of 6.3 years was not different between groups, but this trial was not designed to test the effectiveness of blood pressure lowering in a population.

The effect of blood pressure lowering on covert cerebrovascular disease has also been studied, with observational studies reporting the association of antihypertensive therapy with reduction in risk of developing white matter hyperintensities (57). In two recent RCT sub-studies, blood pressure lowering was associated with a reduction in the rate of progression of WMH. In the INFINITY (58) (*n* = 199) and SPRINT-Mind (59, 60) (*n* = 670) trials, blood pressure lowering was associated with a smaller percentage change in white matter hyperintensity volume, and, in the SPRINT-Mind trial, a greater decrease in brain volume, based on comparison of MRI brain imaging at baseline and follow-up.

There is an absence of clear information on what, if any, is the best antihypertensive medication class for prevention of dementia or cognitive decline. Randomized controlled trials reporting cognitive outcomes have employed diverse antihypertensive agents, including ACE inhibitors (61), diuretics (62), calcium channel blockers (63) and angiotensin receptor blockers (64). Two recent systematic reviews evaluating the effect of particular classes of antihypertensive drugs on cognitive outcomes did not report a larger benefit of any one class of antihypertensive drug over another (65, 66). However, these meta-analyses were underpowered to determine between-class effects of antihypertensive drugs. A recent Phase II trial (*n* = 176) reported superiority of candesartan vs. lisinopril in mean change in some neurocognitive tests over 12 months (67). However, there are no large randomized controlled trials comparing different antihypertensive agents with clinical syndrome of dementia or cognitive impairment as the primary outcome.

## Cognitive Impairment and Other Blood Pressure Parameters

Steep declines in blood pressure in later life, compared to mid-life, are associated with development of cognitive impairment (10, 68). This observation suggests that cerebral perfusion plays a significant role in development of dementia, and that the association between blood pressure and development of cognitive impairment is not linear and may well be *J* or even *U*-shaped and depends on age (69, 70). The Chinese Longitudinal Healthy Longevity survey reported that a systolic blood pressure range of 130–150 mmHg was associated with the lowest risk of cognitive impairment in adults over 80 years (71). The association of lower blood pressure and risk of dementia in older age may be due to reverse causation.

Orthostatic hypotension is a risk factor for dementia, associated with a 26% relative increase in risk. It is common in older adults and can affect cognition through a number of mechanisms. First, neurodegeneration of brain regions responsible for cognitive function may also be involved in regulation of cardiovascular activities leading to orthostatic hypotension cognitive impairment. Second, orthostatic hypotension can cause poor frontal lobe perfusion which can affect executive function. Third, low cerebral blood flow can cause subcortical infarction and ischemic demyelination (72).

Blood pressure variability (BPV) is increasingly being recognized as having a significant role in target organ damage (73). Several longitudinal studies have also reported that BPV is significantly associated with increased risk of cognitive impairment and dementia (11, 74–76). Higher BPV between sequential visits was associated with a higher long-term risk of dementia in the Rotterdam study which was most pronounced when BPV was measured 15 years before the diagnosis of dementia (77). Another study showed that increased day to day BPV is also associated with higher risk of dementia over 5 years but the long-term risk of dementia in relation to day to day BPV is unknown (76). The precise biological mechanism relating blood pressure variability with cognitive impairment is incompletely understood, but likely related to subclinical ischemic changes in brain, hypoperfusion and hypotension, endothelial dysfunction and inflammation all playing a role. Reverse causation may also have a role where there is BPV due to autonomic dysfunction associated with dementia syndromes (78).

In clinical practice, use of antihypertensive agents and regimens associated with least blood pressure variability and orthostasis may be more appropriate for older patients with hypertension (79).

## Conclusion/Future Directions

Hypertension is an important modifiable risk factor for cognitive impairment and dementia. Evidence from randomized controlled trials suggests a 7–11% relative risk reduction in the incidence of dementia with antihypertensive therapy. While none of these trials included cognitive outcomes as the primary outcome measure, it is unlikely that large definitive trials will be completed, as antihypertensive agents are indicated for primary prevention of cardiovascular disease in individuals with hypertension. Future studies are needed to determine the optimal blood pressure target, especially in older adults and those with orthostatic hypotension. Additional research is also required to determine which antihypertensive agents, and regimens, are optimal for maintaining cognitive health. Of greater importance, however, is the need for improved detection and treatment of hypertension in the general public, which is expected to translate into meaningful gains in lowering the global burden of dementia.

## Author Contributions

MC and MO'D both conceptualized the paper, designed the figures and tables, and wrote sections of the first draft of the manuscript. Both authors contributed to subsequent manuscript revision and refinement, read, and approved the submitted version.

## Conflict of Interest

The authors declare that the research was conducted in the absence of any commercial or financial relationships that could be construed as a potential conflict of interest.

## Publisher's Note

All claims expressed in this article are solely those of the authors and do not necessarily represent those of their affiliated organizations, or those of the publisher, the editors and the reviewers. Any product that may be evaluated in this article, or claim that may be made by its manufacturer, is not guaranteed or endorsed by the publisher.
